# Role of Temperate Bacteriophage ϕ20617 on *Streptococcus thermophilus* DSM 20617^T^ Autolysis and Biology

**DOI:** 10.3389/fmicb.2018.02719

**Published:** 2018-11-09

**Authors:** Stefania Arioli, Giovanni Eraclio, Giulia Della Scala, Eros Neri, Stefano Colombo, Andrea Scaloni, Maria Grazia Fortina, Diego Mora

**Affiliations:** ^1^Department of Food Environmental and Nutritional Sciences, University of Milan, Milan, Italy; ^2^Sacco Srl, Cadorago, Italy; ^3^Proteomics and Mass Spectrometry Laboratory, Istituto per il Sistema Produzione Animale in Ambiente Mediterraneo, National Research Council, Naples, Italy

**Keywords:** *Streptococcus thermophilus*, bacteriophage, bioenergetics, biofilm, heat-resistance

## Abstract

*Streptococcus thermophilus* DSM 20167^T^ showed autolytic behavior when cultured in lactose- and sucrose-limited conditions. The amount of cell lysis induced was inversely related to the energetic status of the cells, as demonstrated by exposing cells to membrane-uncoupling and glycolysis inhibitors. Genome sequence analysis of strain DSM 20617^T^ revealed the presence of a *pac*-type temperate bacteriophage, designated Φ20617, whose genomic organization and structure resemble those of temperate streptococcal bacteriophages. The prophage integrated at the 3′-end of the gene encoding the glycolytic enzyme enolase (*eno*), between *eno* and the lipoteichoic acid synthase-encoding gene *ltaS*, affecting their transcription. Comparative experiments conducted on the wild-type strain and a phage-cured derivative strain revealed that the cell-wall integrity of the lysogenic strain was compromised even in the absence of detectable cell lysis. More importantly, adhesion to solid surfaces and heat resistance were significantly higher in the lysogenic strain than in the phage-cured derivative. The characterization of the phenotype of a lysogenic *S. thermophilus* and its phage-cured derivative is relevant to understanding the ecological constraints that drive the stable association between a temperate phage and its bacterial host.

## Introduction

Bacterial autolysis results from the degradation of peptidoglycan, the major structural component of the bacterial cell wall, through the action of peptidoglycan hydrolases. In several lactic acid bacteria such as *Lactococcus lactis* and *Lactobacillus* (Valence and Lortal, 1995; Buist et al., 1997), an autolytic phenotype resulted from the uncontrolled action of bacterial peptidoglycan hydrolases.

Among the lactic acid bacteria species, *Streptococcus thermophilus* autolytic strains have rarely been detected (Sandholm and Sarimo, 1981; Thomas and Crow, 1983; Husson-Kao et al., 2000a,b), and autolysis was often associated with a lysogenic phenotype. In *S. thermophilus* type strain DSM 20617^T^, the autolytic phenotype has been correlated with the induction of a leaky prophage (Husson-Kao et al., 2000a,b), even though the phage was not characterized. In some *S. thermophilus* strains, prophage excision occurs under stressed culture conditions with limiting lactose and sucrose concentration (Thomas and Crow, 1983), and cell lysis was triggered by NaCl, chloroform and mitomycin C. It was hypothesized that lysis might be induced by energy starvation and the subsequent dissipation of the proton motive force (Husson-Kao et al., 2000b). It was also proposed that prophage lytic proteins, namely 31-kDa endolysin and holins, would cause lysis and that the scheduling of this lysis would be controlled by mechanisms governing the activity the holins present in small amounts in the membranes during growth.

Although the autolytic phenotype of the *S. thermophilus* strains is linked to their lysogenic character, and the phenotype is observed under unfavorable physiological conditions, cell lysis does not appear to result from massive prophage excision in response to stress, but rather from an incomplete prophage repression (Husson-Kao et al., 2000a,b). Lysis occurs at the end of the exponential growth phase, resulting in a typical bell-shaped growth curve. More recently, *S. thermophilus* DSM 20617^T^ was investigated to characterize the expression of superinfection exclusion proteins from the Ltp type, a mechanism by which a prophage residing in a host cell prevents the infection of the lysogenic host cell by other phages by blocking their DNA injection (Ali et al., 2014).

The autolytic phenotype of the strain DSM 20617^T^ was of particular benefit in the context of specific immunotherapy because, once ingested, bacterial cells can get to autolysis in the intestine. As a consequence, recombinant *S. thermophilus* cells expressing the rBet v 1 allergen release intracellular molecules and enzymes, including recombinant allergens, for the treatment of allergic disorders in humans (Petrarca et al., 2014).

In this study, the autolytic phenotype of *S. thermophilus* DSM 20617^T^ was further investigated, with a focus on the factor triggering cell lysis, and on evaluating the cell wall integrity at different stages of growth. Moreover, we performed a genetic characterization of the prophage ϕ20617 for the genome of *S. thermophilus* DSM 20617^T^. Comparative experiments, that were performed between *S. thermophilus* DSM 20617^T^ and the phage-cured derivative were also performed to describe the impact of ϕ20617 on the physiology of lysogenic bacterium.

## Materials and Methods

### Bacterial Strains, Growth Conditions, and Reagents

Wild-type *S. thermophilus* DSM 20617^T^ and its derivative, A33, were maintained in M17 broth (Difco Laboratories, Detroit, MI, United States) containing 2% (wt/vol) lactose. Growth curves were evaluated at 37°C in M17 containing different concentration of lactose or sucrose in the presence or absence of the glycolytic inhibitor sodium oxamate (Sigma-Aldrich, Milan, Italy), and at different inoculum concentrations. The cell density (O.D. _600nm_) was measured manually using a SmartSpec^TM^ Plus spectrophotometer (BioRad Laboratories, Milan, Italy). When needed, bacterial were grown in 384-well plates that had been filled by an automatic liquid handling system (EpMotion, Eppendorf, Italy) to a final volume of 80 μl per well. In this case, the microbial growth was monitored automatically with a spectrophotometer (Biotek, Winoosky, VT, United States) that was programmed for 145 readings (O.D. _600nm_) every 10 min, for 24 h, at 37°C. At the end of the incubation, the growth curve and the maximum cell density were obtained using the software program Gene5 (Biotek, Winoosky, VT, United States).

### Induction Assay

To evaluate the role of the energetic status of *S. thermophilus* DSM 20617^T^ in the cell lysis, a culture was grown in M17 (2% wt/vol lactose), at 37°C, until reaching O.D. _600__nm_ of 0.8. The cell biomass was collected by centrifugation, washed in saline solution (NaCl 9 g/L) and inoculated in fresh M17 medium with and without 2% (wt/vol) lactose, and containing the protonophore gramicidin (100 μM) or the lactate dehydrogenase/glycolytic inhibitor sodium oxamate (0.4 M) (Sigma-Aldrich, Milan, Italy) (Arioli et al., 2010). The changes in the optical densities were measured every 15 min over 10 h at 37°C using a SmartSpec^TM^ Plus (BioRad Laboratories, Milan, Italy) spectrophotometer.

### Bacterial DNA Extraction, Sequencing and Analysis

*Streptococcus thermophilus* DSM 20617^T^ was grown in M17 (2% wt/vol lactose) for 16 h, at 37°C. The cells collected by centrifugation were subjected to total DNA extraction using an alkaline cell lysis protocol (Manachini et al., 1985). The purified DNA was then sequenced using an Ion Torrent PGM platform (Life Technologies, Carlsbad, CA, United States). A genomic library was constructed using 1 μg of genomic DNA, an Ion Xpress Plus fragment library kit by employing Ion Shear chemistry, according to manufacturer’s instructions. After a dilution to 2.66 × 10^7^ molecules/μL, 4.5 × 10^8^ molecules were used as the template for clonal amplification on Ion Sphere particles during emulsion PCR, as indicated in the Ion Xpress Template 400 kit manual. The quality of the amplification was estimated, and the amplification product was then loaded onto an Ion 316 chip; it was subsequently sequenced using 125 sequencing cycles according to the Ion Sequencing 400 kit user guide. A total of 125 sequencing cycles produced an average read length of approximately 400 nucleotides. MEGAnnotator software (PMID: 26936607) was used to assemble of the genome. Open Reading Frame finder^1^ and RAST Server (Aziz et al., 2008) were used to predict the potential coding regions. The prediction was confirmed by visual inspection using criteria such as the starting codon (ATG, TTG, or GTG), the presence of at least 30 amino acid (aa) and a ribosomal binding site (RBS) similar to a standard Shine-Dalgarno sequence (AAAGGAGGTGA) (Guglielmotti et al., 2009). Translated ORF products were compared with known sequences using BLASTp (Altschul et al., 1997); PSI-BLAST and InterProScan at EMBL-EBI^2^ were used to search for more distant homologous proteins and conserved domains, respectively, when any significant similarity was found by the BLAST searches. The theoretical molecular mass (MM) and isoelectric points (pI) values of the phage proteins were obtained using the ProtParam tool^3^. tRNAs were identified using the tRNAscan-SE server (Lowe and Eddy, 1997) and confirmed using the ARAGORN program (Laslett and Canback, 2004). Virulence Factor Database (Chen et al., 2012) together with DBETH (Chakraborty et al., 2012) were used to search the virulent factors. The genome organization and the proteome of phage 20617 were compared to those of another 13 *Streptococcus* lytic and temperate phages as follows: ALQ13.2 (FJ226752), 858 (NC_010353), O1205 (NC_004303), 2972 (NC_007019), Sfi11 (NC_002214), 7201 (NC_002185), Abc2 (NC_013645), 5093 (NC_012753), DT1 (NC_002072), Sfi19 (NC_000871), Sfi21 (NC_000872), TP-778L (NC_022776), and TP-J34 (NC_020197).

### Quantification of the Excision Events in *S. thermophilus* Population

The dynamics of the excision events (i.e., cells without the prophage in the chromosome) during the growth of *S. thermophilus* DSM 20617^T^ was evaluated by qPCR using the primer set IntR1-ExcR, which were targeted to the *eno* gene and in the intergenic region between the *eno* and *ltaS* genes, respectively (Figure 4). The amplified region encompassed the integration site of ϕ20617. Preliminary experiments showed that the *S. thermophilus* DSM 20617^T^ population is genetically heterogeneous because the PCR signal associated with phage excision was detected together with the PCR signal generated by the primer set IntR1-IntR2 (Supplementary Figure S4) that was targeted to the *eno* gene (on the bacterial chromosome) and the *int* gene (on the phage chromosome), respectively, and designed to monitor the integration of ϕ20617 in the bacterial chromosome. To this end, 1 mL of broth culture was sampled during bacterial growth, and the cells were collected by centrifugation and subjected to DNA extraction, as described in Arioli et al. (2007). qPCR was performed using 5 μL of DNA solution (DNA 5 ng/μL) in a total volume of 20 μL, by using the EvaGreen^TM^ kit (BioRad, Laboratoires, Milano, Italy) and following manufacturer’s recommendations. PCRs were performed in triplicate and run on a CFX96 instrument (BioRad, Laboratoires, Milano, Italy). Data were recorded as threshold cycles (*C_T_*), expressed as the mean ± standard deviations, and analyzed using BioRad CFX Manager^TM^ software. A calibration curve that reported the *C_T_* *vs*. number of cells, was obtained and expressed as the Log_10_ Fluorescent Units (FU). A quantified suspension of the *S. thermophilus* A33 strain (ranging from 10 to 10^6^ FU) was subjected to DNA extraction as described before, and the DNA was used as a template in qPCR assays with IntR1-ExcR as the primer set (the calibration curves obtained for *S. thermophilus* A33 are shown in Supplementary Figure S5). The count of the *S. thermophilus* cells was performed by flow cytometry as described later, and it is expressed as FUs per mL (FU/mL). The excision events were expressed as the % of the total population. The excision events in FU/mL were calculated from the *C_T_* using the calibration curve, whereas the total population of the culture sample was measured directly by flow cytometry and expressed as FU/mL.

### Transcription Analysis

The total RNA was extracted from *S. thermophilus* cells that were collected during exponential stationary phase of growth in M17 (2% wt/vol lactose or 1% wt/vol sucrose), at 37°C according to Arioli et al. (2010). A 1 μg quantity of RNA was used for cDNA synthesis by iScript^TM^ cDNA Synthesis (BioRad, Laboratoires, Milano, Italy). RT-qPCR was performed using 5 μl of cDNA solution in a total volume of 20 μL using the EvaGreen^TM^ kit (BioRad, Laboratoires, Milano, Italy). The expression levels *ureC*, the primary subunit of urease; *eno*, coding for the glycolytic enzyme enolase; *ltaS*, coding for lipotheichoic acid synthase; and the prophage genes coding for holin, lysin and a phage tail protein (ORF 55, 56, and 48) were normalized using *pgk*, which codes for phosphoglycerate kinase, as the reference gene. For each condition, the measures were performed in triplicate with cDNA synthesized from two independent RNA samples. The real-time PCR was performed using the EvaGreen PCR master mix (BioRad, Laboratoires, Milano, Italy) as recommended. PCRs were performed in triplicate and run on a CFX96 instrument (BioRad, Laboratoires, Milano, Italy). Data were recorded as threshold cycles (*C_T_*), expressed as the mean ± standard deviation, analyzed using the BioRad CFX Manager^TM^ software and expressed as normalized expression (ΔΔ*C_T_*) ± the standard error of the mean. The primer sets used in the real-time PCR experiments are shown in Supplementary Table S3.

### Flow Cytometry Measurements

The cell suspensions were diluted in sterile filtered (0.2 μm) water and stained with a cell-permeant SYBR green I (1X) (Sigma-Aldrich, Milan, Italy) or to a double staining using SYBR green I and propidium iodide (PI) (Arioli et al., 2019). After incubation at room temperature for 15 min, the labeled cell suspensions were diluted to reach approximately 10^6^ events/ml, and they were analyzed by flow cytometry. Cell suspensions that were prepared as described above were analyzed using the flow cytometer, according to Arioli et al. (2019) and the obtained data were analyzed using BD Accuri^TM^ C6 software version 1.0 (BD Biosciences, Milan, Italy). To detect cell debris as a consequence of the autolytic phenotype, the SYBR green I stained cell suspension was subjected to DNAse I (SIGMA-Aldrich) treatment according to the manufacturer instruction. To follow the cell-wall turnover and biosynthesis, cells growing at 37°C in M17 in the presence of different concentrations of sucrose were subjected to BODIPY-FL vancomycin staining. BODIPY-FL vancomycin binds specifically to nascent peptidoglycan. To this end, an appropriate dilution of *S. thermophilus* cells in saline solution (NaCl 9 g/L) was incubated with 1 μg/mL (final concentration) of BODIPY-FL vancomycin mixed with an equal quantity of unlabeled vancomycin, for 15 min, as previously described (Daniel and Errington, 2003). After incubation, the samples were analyzed by flow cytometry. BODIPY-FL vancomycin fluorescence intensity of stained cells was recovered in the FL1 channel.

### Evaluation of Cell Wall Integrity

The cell-wall integrity of the wild-type and A33 derivative mutant were evaluated using the following approaches: (i) the sensitivity to lysozyme/SDS treatment and (ii) cells fragility in response to vortex mixing. For the evaluation of the lysozyme/SDS treatment, cells were grown in M17 containing 2% wt/vol lactose, at 37°C, and collected at exponential growth phase (O.D. _600nm_ 0.5) or late stationary phase (O.D. _600nm_ 2.0). The cells from 1 mL of culture were collected by centrifugation at 10,000 ×*g* for 3 min, washed in 0.1 M Tris-HCl buffer pH 8, suspended in 10 mL of the same buffer containing lysozyme (0.1 mg/mL) and incubated for 4 h, at 37°C. After the incubation, the suspension was treated with 0.015% (w/v) SDS, mixed gently by inversion, and incubated for 5 min, at room temperature. The lysozyme/SDS sensitivity was expressed as the % decrease in cellular density (O.D. _600nm_) after the enzymatic/SDS treatment compared to the cellular density before the treatment.

The integrity of the cell wall was also assayed by evaluating cell sensitivity in response to vortex mixing stress in 1.5 mL-tubes (Eppendorf, Milan, Italy). The cell integrity was evaluated by collecting the biomass as described before. The collected biomass was gently suspended in 500 μL of Tris-HCl buffer, subjected to a vortex mixing for 2 min and centrifuged at maximum speed (15000 ×*g*) for 3 min, and the supernatant was recovered to evaluate its β-galactosidase activity. To determine the β-galactosidase activity, 100 μL of supernatant was added to 900 μL of Tris-HCl buffer containing 2-nitrophenyl-β-D-galactopyranoside (0.2 mg/mL) (Sigma-Aldrich, Milan, Italy). The measurements of the β-galactosidase activity were performed at 37°C, by monitoring O.D. _420nm_ with a microplate-reader (MicroWave RS2, Biotek, United States) that was programmed to read a set of 60 repetitions at intervals of 30 s each. The β-galactosidase activity was expressed in mO.D. _420nm_/min, as the mean of four independent determinations.

### Cell Adhesion to Solid Surface

The adhesion to solid surfaces, which is a property that was reportedly to be linked to peptidoglycan breaks in *L. lactis* (Mercier et al., 2002), was evaluated as previously described (Djordjevic et al., 2002), with minor modifications. *S. thermophilus* strains were cultured in M17 broth (2% wt/vol lactose) in six-wells PVC tissue plates (VWR, Milan, Italy) for 24 h, at 37°C. After a 24 h incubation, the medium was removed from the wells and the microtiter plate wells were washed five times with sterile distilled water to remove any loosely associated bacteria. The plates were air-dried for 45 min, and each well was stained with 150 μL of 1% crystal violet solution in water, for 45 min. After staining, the plates were washed with sterile distilled water five times. At this point, the biofilms formed by *S. thermophilus* cells were visible as a purple color that formed on each well. The quantitative analysis of the biofilm production was performed by adding 200 μL of 95% v/v ethanol to destain the cell biomass that had adhered to the wells. Two hundred microliters from each well were transferred to a 96-well microtiter plate, and the level of the crystal violet present in the de-staining solution was measured at 595 nm. Adhesion levels of *S. thermophilus* cells were reported as mO.D. _595nm_. The adhesion of the *S. thermophilus* to solid surfaces was also determined using a bioluminescence approach. *S. thermophilus* DSM 20617^T^ and A33 phage-cured derivative were transformed by electroporation with the vector pCSS945 vector, which was carrying a *lucGR* gene coding for a Jamaican click beetle luciferase (Loimaranta et al., 1998) as described previously (Arioli et al., 2010). To evaluate cells adhesion on solid surfaces, the strains harboring the pCSS945 vector were grown in M17 broth containing 2% (wt/vol) lactose and chloramphenicol (4 μg/mL) for 18 h, at 37°C, in 24-well tissue culture plates (PerkinElmer, Milan, Italy). After growth, the cell cultures were removed from the wells to evaluate their optical density (O.D. _595nm_) with microplate reader M680 (Bio-Rad Laboratories, Hercules, CA, United States). The wells were washed twice with 1 mL of sterile distilled water, filled with 1 mL of M17 broth containing 2% (w/v) lactose and chloramphenicol (4 μg/mL) and incubated for 30 min, at 37°C. After incubation, 50 μl of 1 mM D-luciferin (0.1 M sodium citarte buffer, pH 5.0) was added, the plate was subjected to light emission measurements with a Victor3 luminometer (PerkinElmer, Milan, Italy) that was programmed to read 24 repetitions at intervals of 300 s, at 37°C.

### Growth of *S. thermophilus* at Different Temperatures, and Survival of *S. thermophilus* in Response to Heat-Treatment

*Streptococcus thermophilus* strains were inoculated (10^7^ events/ml) in M17 broth (2% wt/vol lactose), dispensed (100 μL) in PCR tubes and incubated 18 h in a thermal-cycler (Mastercycler Nexus Gradient, Eppendorf, Milan, Italy) with a gradient temperature ranging from 40°C to 60°C, to incubate each strain at 40, 40.5, 41.0, 42.0, 43.0, 44.5, 46.0, 47.0, 48.0, 49.0, and 50.0°C, in duplicate. After incubation, the bacterial growth was measured spectrophotometrically (O.D. _600nm_). The survival of *S. thermophilus* strains to heat treatment was tested as following. *S. thermophilus* cultures were grown in M17 broth (2% wt/vol lactose), collected at exponential phase of growth (O.D. _600nm_ 0.5) by centrifugation, suspended in saline solution (9 g/L NaCl), and standardized to a cell concentration of 2 10^9^ events/mL by flow cytometry counting. Standardized cell suspensions, were dispensed (100 μl) in PCR tubes and incubated for 10 min at the following temperatures: 54.0, 56.0, 59.0, 61.0, 64.0, 66.5, 68.0, 69.5, and 70.0°C using a thermal-cycler as described above. After heat treatment, samples were rapidly cooled and subjected to serial dilution and plate counting on M17 (2% wt/vol lactose).

### Colony Description, Culture and Cell Phenotype

The colony dimensions of *S. thermophilus* DSM 20617^T^ and its derivative A33 were evaluated in M17 (2% wt/vol lactose) using a stereo microscope (Zeiss, Italy). To confirm the hypothesis that strain DSM 20617^T^ had a reduced chain length due to the activity of phage ϕ20617 lysin compared to its derivative A33, we set up a sedimentation experiment as described by Mercier et al. (2002). We evaluated the differential sedimentation rates of the bacteria according to their chain-forming and or cell size in semi-liquid medium (i.e., liquid M17-lactose medium containing 0.15% wt/vol agarose). To this end, a drop of DSM 20617^T^ or A33-saturated culture was deposited at the surface of the semi-liquid medium and the culture growth was measured as its distance from the surface, after 18 h of incubation at 37°C. To confirm the differences in cell morphology between strain DSM 20617^T^ and A33, cells were collected from the semi-liquid cultures and photographed under a phase-contrast microscope.

## Results and Discussion

### The Autolytic Behavior of *S. thermophilus* DSM 20617^T^ Is Triggered by Cell Bioenergetics

The autolytic phenotype in *S. thermophilus* is quite rare, and in *S. thermophilus* DSM 20617^T^ (ATCC19258, CNRZ1358, NCDO573, and WDCM00134) it was found to depend on the induction of a leaky prophage (Sandholm and Sarimo, 1981; Thomas and Crow, 1983; Husson-Kao et al., 2000a,b), here designed Φ20617.

**FIGURE 1:**
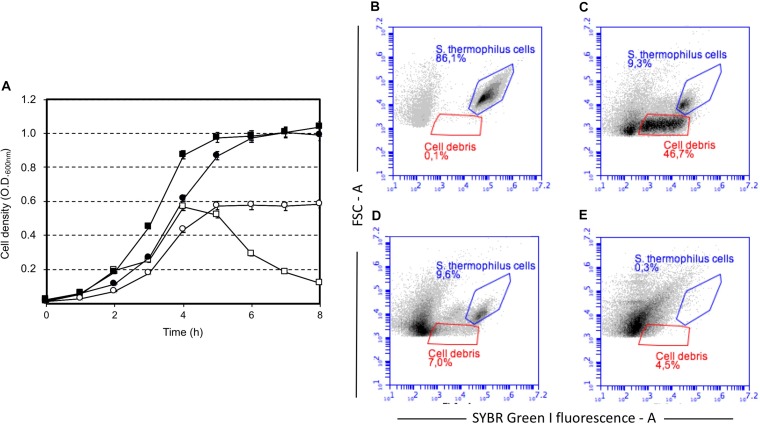
Growth of *Streptococcus thermophilus* DSM 20617^T^ (squares) and its derivative A33 (circles) strain in M17 broth in the presence of sucrose, and lactose. **(A)** Growth of *S. thermophilus* strains in the presence of sucrose at a final concentration of 0.2% (white symbols) and 1% (black symbols). Strain DSM 20617^T^ (autolytic) (white and black squares), strain A33 (non-autolytic) (white and black circles). Flow cytometry of SYBR Green I stained cells of *S. thermophilus* DSM 20617^T^ grown in M17 (0.2 % wt/v sucrose). **(B)** Cells after 4 h of growth at 37°C (blue gate). **(C)** Cells and cell debris (red gate) after 8 h of growth at 37°C. **(D)** Cells and cell debris (red gate) after 8 h of growth at 37°C treated with DNAse. **(E)** M17 broth SYBR Green I stained.

**FIGURE 2:**
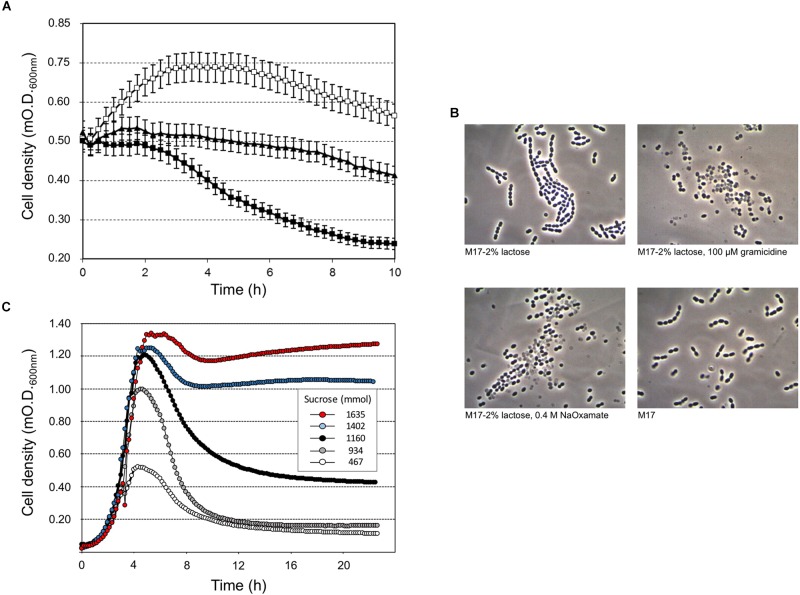
*Streptococcus thermophilus* DSM 20617^T^-induced lysis **(A)** and cell morphology **(B)** after 10 h of incubation at 37°C, in the presence of gramicidin (black triangles), membrane uncoupling reagent, or sodium oxamate (black squares), which is a lactate dehydrogenase inhibitor. Cells grown in M17 (2% wt/vol lactose) at 37°C were collected at O.D. 0.55, and washed and suspended in fresh M17 at 37°C, without lactose (white squares) in the absence/presence of gramicidin or sodium oxamate. Error bars represent the standard deviations based on three replicates. **(C)** *S. thermophilus* DSM 20617^T^ was grown in microtiter plates using increasing concentrations of sucrose (0.05–3%) in presence of sodium oxamate. The growth curves were extrapolated as example for a total of 384 different culture conditions as reported in Supplementary Figure S3.

Our study highlighted that cell lysis was triggered by low sugar concentrations, that’s 0.5 or 0.2 (wt/v) lactose or sucrose, respectively, and higher lactose or sucrose concentrations prevented cell autolysis (Figure 1). Moreover, sucrose was more effective than lactose at cell lysis induction (Figure 1A, Supplementary Figure S1). Flow cytometry analysis of *S. thermophilus* culture grown at low sucrose concentration revealed the relevant decrease in cell concentration in favor of the formation of cell debris when lysis occurred (Figure 1B–D). During the standard culture procedures of strain DSM 20617^T^, we fortuitously observed one colony in the M17 agar Petri plates that was characterized by a diameter smaller than the majority of the other colonies (Supplementary Figure S2). From that colony, we isolated a derivative cured strain, named A33, which maintained the small colony phenotype and lost its autolytic behavior (Figure 1). Giving that the lysis of strain DSM 20617^T^ was linked to the sugar concentration of the medium, we further investigated the involvement of the energetic metabolism in the autolytic phenotype. For this purpose, cells collected during exponential growth phase were harvested and then suspended in fresh M17 medium without lactose or with lactose but in the presence of gramicidin or sodium oxamate. Gramicidin is an ionophore that dissipates the membrane ion gradient between the cytoplasm and the extracellular environment; sodium oxamate is a glycolytic inhibitor, analogous to pyruvate, and it blocks lactate dehydrogenase activity, therefore inhibiting the energetic metabolism of *S. thermophilus* cells (Arioli et al., 2010). Cells that were exposed to sodium oxamate started to lyse after 2 h of incubation, whereas only moderate lysis was observed in presence of gramicidin through 10 h of incubation (Figure 2A). Complete cell lysis was not observed, but an interesting reduction in the chain length (Figure 2B) was clearly detectable for those cells that, although in presence of lactose, were exposed to sodium oxamate or gramicidin, thus suggesting that a peptidoglycan hydrolase activity was triggered by the energetic stress induced by these molecules. Previously, the control of autolysin activity and energized membrane was reported in *Bacillus subtilis*. In specific, when cells were deprived of a carbon source the proton motive force collapsed, and the autolysis occurred (Joliffe and Streips, 1981).

**FIGURE 3:**
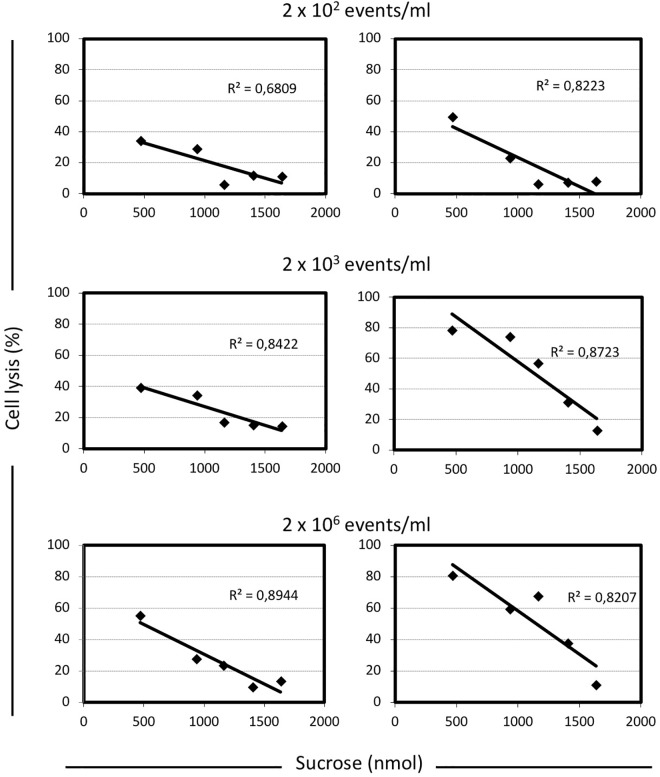
Correlation between the level of cell lysis and the total amount of sucrose in M17 broth, in presence or absence of sodium oxamate. For each condition analyzed here, the cell lysis was calculated as follow: [(maximum cell density reached – cell density at the end of the incubation time)/maximum cell density reached] × 100. The inoculum concentration was indicated. Linear regression equations and the *R*^2^-values are indicated in each graph. Shaded graphs represent the data obtained when M17 was supplemented with 10 mM sodium oxamate.

To better understand the relationship between lysis, sugar concentrations, and energetic metabolism *S. thermophilus* was cultivated in 384 wells plate using different level of inoculum, in the presence of increasing sucrose concentrations, and in the absence or presence of sodium oxamate, for a total of 384 different culture conditions (Supplementary Figure S3). The resulting data showed the inverse correlation between the level of cell lysis and the amount of sucrose in the medium (Figure 2C). However, the observed inverse correlation between the amount of sugar and cell lysis was also dependent on the inoculum level; indeed, the higher percentage of cell lysis was always obtained when cells were cultured in the presence of sodium oxamate (Figure 3 and Supplementary Table S1). While the triggering effect of low sugar concentrations on *S. thermophilus* cell lysis was described previously (Sandholm and Sarimo, 1981; Thomas and Crow, 1983; Husson-Kao et al., 2000a,b), the sucrose concentration-dependent cell lysis, presented in this study is novel. Moreover, the use of the glycolytic inhibitor sodium oxamate and its triggering of autolysis allowed us to link this phenotype to the decreased of cell energetic level (Figures 2A, 3). The involvement of the energetic metabolism in cell autolysis was previously observed in *L. lactis* (Riepe et al., 1997), in *Enterococcus faecalis* (Shockman et al., 1961) and in *B. subtilis* (Joliffe and Streips, 1981), but it was never related to a lytic cycle of a temperate prophage.

### The *S. thermophilus* DSM 20617^T^ Genome Hosts a *pac* Type-Temperate Prophage

A draft genome sequence of strain DSM 20617^T^ allowed for the identification of a contig containing the entire genome of temperate prophage 20167 (accession number NC_023503). Prophage 20167 is 41,007 bp long with a %GC of 40%, which are features that are common to other *S. thermophilus* phage genomes as well as the %GC of the species (Guglielmotti et al., 2009). As reported in Table S2, 61 ORFs were predicted in the genome, and they contained 30 or more ORFs that were grouped into 4 typical distinct modules (lysogeny, replication, morphogenesis and lysis). Out of 61 predicted proteins, functions were attributed to 29 of them (47%), while all the proteins matched up with similar sequences found in both *Streptococcus* phages and bacteria. No tRNA of any virulence factor was found along the genome. Worth mentioning was the observed similarity of ORF2 to superinfection exclusion lipoprotein (sie) of *Streptococcus* phage TP-778L. This protein is well known to be involved in preventing the entry of foreigner phage DNA molecules into the cell, which permits both the cell and temperate phage to overcome destruction during lytic phage replications (Ali et al., 2014). Moreover, five extra genes located between the lysis gene (ORF56) and the right attached site were found. This module, which is known as the lysogenic conversion, was also described in other *S. thermophilus* phages (Ventura et al., 2002a,b), as well as in pathogenic *Streptococcus* species such as *Streptococcus* *pyogenes* and in *Staphylococcus aureus* prophages. The role of these genes (which normally range from one to six) is still unclear but they were hypothesized to be involved in lysogenic conversion phenotypes, increasing the ecological fitness of the lysogen to further their evolutionary success; through transcriptomic analysis, researchers also demonstrated their expression in the lysogenic state (Ventura et al., 2002b), confirming their importance to lysogeny. In particular, these genes encode different virulence factors in pathogenic bacteria such as toxins, superantigens, mitogenic factors and DNAses (Kaneko et al., 1998; Desiere et al., 2001; Ferretti et al., 2001). For temperate phage 20167, a lower GC content for this region (35%) compared to the rest of the genome (40%) strongly highlighted its foreign origin, which could have occurred during a faulty prophage excision from an unusual bacterial host.

Comparative analysis of the genetic organization and content of prophage 20617 with other temperate and lytic *S. thermophilus* phages confirms the relatedness of phage 20617 to other *Streptococcus* phages, in particular to those belonging to *pac* species in which the phages are grouped (Mahony and van Sinderen, 2014). This high similarity also permitted us to identify the *pac* as the mechanism that was used when genomes were inserted into capsids during phage replication. This hypothesis was also supported by the presence of three major structural proteins that are coded by ORF36, 38 and 39 (Supplementary Table S2).

Genes belonging to the morphogenesis and lysis modules of phage 20617 are well conserved, compared to lytic *pac* phage ALQ13.2 (Guglielmotti et al., 2009), with an amino acid identity ranging from 81 to 88%, while some replication genes showed homology (for an amino acid identity of 87%) with lytic cos phage 7201. Lysogenic genes are instead similar (85–90% identity) to lytic *cos* phage 5093, representing the third *Streptococcus* phage species outside of the cos-type/pac-type grouping scheme and hypothesized to be the ancestral link between phages infecting *S. thermophilus* and its non-dairy ancestor (Mills et al., 2011).

### Excision Dynamics of Phage 20617 and Transcription Analysis

**FIGURE 4:**
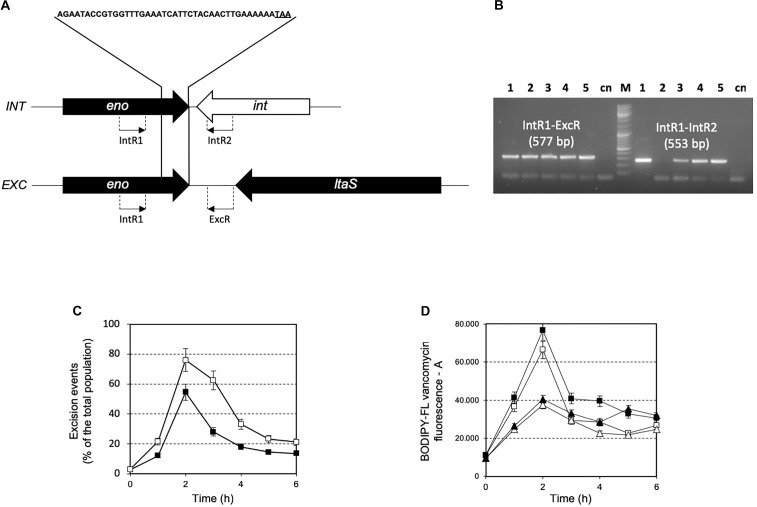
**(A)** Schematic representation of integration (*INT*) and excision (*EXC*) structure in the *S. thermophilus* DSM 20617^T^ genome. The 43 bp sequence recombination site is indicated. *eno*, enolase coding gene; *ltaS*, lipoteichoic acid synthase coding gene; *int*, phage integrase coding genes. The location of PCR primers designed for the specific amplification and identification of the integration and excision structure are indicated. **(B)** PCR amplification for the identification of integration and excision structures on genomic DNA extracted from *S. thermophilus* DSM 20617T and A33 phage-cured strains. Line 1, 3, 4, and 5 PCR product obtained using as template DNA from *S. thermophilus* DSM 20617^T^ grown in M17 lactose 1%, 0.5%, sucrose 1%, and sucrose 0.2%, respectively. Line 2, PCR product using as template DNA from *S. thermophilus* A33 phage-cured. M, molecular weight marker. cn, PCR negative control. Primer sets and the relative expected dimension of PCR fragment are reported. Dynamics of excision events **(C)** and BODIPY-FL vancomycin fluorescence **(D)** during the growth of *S. thermophilus* DSM 20617^T^ in M17 containing 0.2% (white symbols) and 1% (wt/vol) (black symbols) sucrose. *S. thermophilus* wild-type (squares), and A33 phage-cured (triangles). Error bars represent the standard deviation based on three replicates.

The prophage was integrated at the 3′-end of the gene coding for the glycolytic enzyme enolase (*eno*). Phage integration generated a duplication of the recombination site as identified in a 43 bp AT-rich region (Figure 4A), that is located precisely at the 3′-end of the *eno* gene, and upstream of the chromosomal gene coding for lipoteichoic acid synthase (*ltaS*). While the 43 bp integration sequence shares common features with other known integration sequences of temperate bacteriophages (Stanley et al., 1997), to our knowledge, the genomic localization of the integration site represents a novelty. Based on the genome sequencing, a PCR assays were performed using primers targeted to the *ptf* gene coding for the putative phage tail fiber (ORF 48 in Supplementary Table S2). The molecular analysis clearly showed that non-autolytic A33 is a phage-cured derivative strain (Figure 4B). More interestingly, the end-point PCR assay that was designed to identify the prophage-free and prophage-integrated chromosomes highlighted that excision events were also common in the lysogenic host (the wild-type) (Supplementary Figure S4). Similar observations have been reported for ΦO1205, and for the lysogenic *L. lactis* LMN-C3 and UC509 (Lillehaug and Birkeland, 1993; Van de Guchte et al., 1994; Stanley et al., 1997). Therefore, we can hypothesize that this phage could stay in a life cycle called pseudo-lysogeny, remaining as a non-integrated and non-replicating pre-prophage, resembling an episome. This phenomenon occurs when bacterial cells are under nutrient-deprived conditions, until the nutritional status is restored and the phage can enter a lysogenic or lytic life cycle (Feiner et al., 2015). In this context, the dynamics of excision events were evaluated for strains DSM 20617^T^ and A33 by qPCR during the growth in M17 supplemented with sucrose at a concentration stimulating that of cell lysis (0.2%), and at a higher concentration (1%) at which cell lysis was not detectable. Sucrose was chosen as the carbon source in this experiment because it was more effective than lactose at lysis induction. The qPCR analysis showed that excision events increased rapidly during the growth of strain DSM 20617^T^, reaching a maximum of 55% after 2 h of incubation in M17 (1% wt/vol sucrose) and 76% in M17 (0.2% wt/vol sucrose) (Figure 4C), before culture lysis was detectable (Figure 1). Prolonging the incubation time led to a rapid decrease in the percentage of excision events, with higher values in the presence of the lowest sucrose concentration in the medium. These results showed that, during the growth of the autolytic strains, regardless the sugar concentration, a strong phage excision was detectable. Interestingly, at the maximum excision events, we measured the highest values for BODIPY-FL vancomycin fluorescence that were detected in the *S. thermophilus* wild-type (Figure 4D). BODIPY-FL vancomycin is useful for detecting vancomycin binding sites, that is D-Ala-D-Ala on peptidoglycan precursors of the cell wall. Therefore, the increasing of the cells fluorescence due to the binding of the stain to its target should be consistent with the massive peptidoglycan synthesis, as expected during exponential growth phase. Because the BODIPY-FL vancomycin fluorescence was significantly higher in the wild-type compared to the A33 phage-cured strain, we hypothesized that the phage-host interaction could affect peptidoglycan synthesis (Figure 4D). A transcription analysis revealed that the phage genes involved in cell lysis, coding for holin, lysine, and the phage tail fiber (ORF 55, 56, and 48, respectively), were all expressed during exponential and stationary growth phases, independently of the carbon source and its concentration (Table 1). The transcription levels of *eno* gene, whose 3′-end region contained the prophage integration site, and of *ltaS* gene, which is located downstream from the integration site, showed moderate but significant differences between the wild-type and the derivative phage-cured A33, thus indicating that phage integration interfered with bacterial gene transcription. Specifically, the *eno* gene was more highly expressed in the phage-cured strain during stationary phase, whereas *ltaS* showed a higher transcription level during exponential growth phase in the wild-type regardless the carbon source. No *ltaS* gene transcription was detected during stationary phase for autolytic and phage-cured strain. All these data could suggest a bacterium-phage interaction, in which prophages integrated into the bacterial chromosome can regulate bacterial genes expression via phage genome excision (Feiner et al., 2015).

**Table 1:** Transcription analysis of bacterial and prophage genes.

	Normalized fold expression			
Gene target	Sucrose		Lactose	
	DSM 20617^T^	A33	DSM 20617^T^	A33
*ureC*^exp^	1.03 ± 0.20	0.78 ± 0.03	1.00 ± 0.11	1.13 ± 0.11
*ureC*^stat^	0.40 ± 0.06	0.50 ± 0.05	0.42 ± 0.05	0.56 ± 0.02
*eno*^exp^	1.00 ± 0.10	0.99 ± 0.04	0.88 ± 0.13	1.03 ± 0.05
*eno*^stat^	0.81 ± 0.02	1.00 ± 0.01	0.93 ± 0.04	1.33 ± 0.10
*ltaS*^exp^	0.90 ± 0.04	0.68 ± 0.05	1.24 ± 0.10	0.80 ± 0.05
*ltaS*^stat^	nd	nd	nd	nd
ORF 48 (phage tale fiber) ^exp^	1.06 ± 0.04	nd	0.85 ± 0.07	nd
ORF 48 (phage tale fiber) ^stat^	1.00 ± 0.01	nd	0.72 ± 0.03	nd
ORF 55 (holin) ^exp^	1.06 ± 0.07	nd	0.81 ± 0.07	nd
ORF 55 (holin) ^stat^	1.00 ± 0.02	nd	0.89 ± 0.04	nd
ORF 56 (lysin) ^exp^	1.06 ± 0.04	nd	0.96 ± 0.08	nd
ORF 56 (lysin) ^stat^	1.00 ± 0.02	nd	1.03 ± 0.04	nd


*^exp^RT-qPCR assay carried out on RNA isolated from *Streptococcus thermophilus* cells collected in exponential growth phase (O.D. _*600nm*_ 0.5). ^*stat*^RT-qPCR assay carried out on RNA isolated from *S. thermophilus* cells collected in stationary growth phase (O.D. _*600 nm*_ 1.5). *ureC* was used as control gene whose expression is known to be modulated during *S. thermophilus* growth; *ureC* transcription was induced in a pH-dependent way during the exponential phase of growth; it decreased in stationary phase (Mora et al., 2005). Normalized fold expression is expressed as normalized expression (ΔΔ*C_*T*_*) ± standard error of the mean.*

### Prophage Affects *S. thermophilus* DSM 20617^T^ Cell Wall Integrity and Cell Adhesion to Solid Surfaces

**FIGURE 5:**
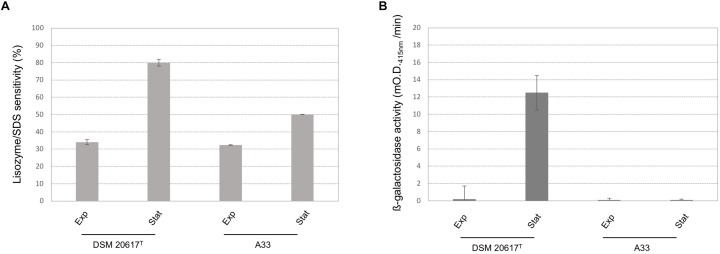
Cell-wall integrity in *S. thermophilus* wild-type and A33 phage-cured derivative. **(A)** Lisozyme/SDS sensitivity was reported as reduction (%) of the cell density (O.D. _595nm_) after the enzymatic treatment. **(B)** ß-galactosidase activity was reported as mO.D. _415nm_ per min. exp = cells collected during the exponential growth phase (O.D. _600__nm_ 0.5). st = cells collected during the stationary phase (O.D. _600__nm_ 1.5). Error bars represent the standard deviation based on three replicates.

Because transcriptional analysis revealed that prophage holin and lysin were expressed during both the exponential and stationary growth phases (Table 1), the cell membrane and cell wall integrity was evaluated. The phospholipid bilayer was not compromised, at least at levels that were not significantly different between the wild-type and the A33 phage-cured strain (Supplementary Figure S6), thus indicating that holin transcription had no effect on the integrity of the cell membrane. As stated by other authors (Neve et al., 2003), we assume that the phage-derived holin stays in a non-active state in the cell membrane and becomes activated with an autolysis-triggering environmental impulse. On the contrary, the peptidoglycan integrity was highly affected in the lysogenic strain, showing higher cell wall fragility compared to the cured A33 strain following lysozyme/SDS sensitivity (Figure 5A) and release of ß-galactosidase activity after a mechanical stress (Figure 5B). Specifically, the cell wall integrity was compromised during stationary growth phase in the wild-type strain; when cells were collected during the exponential phase of growth, no significant differences were observed between the wild-type and the cured A33 strain. In *L. lactis*, a closely related species of *S. thermophilus*, peptidoglycan breaks have been positively correlated with phenotype of adhering to solid surfaces, together with an increase in biofilm formation (Mercier et al., 2002). Therefore, the adhesion phenotype was measured in the wild-type and A33 strains and also in their derivative recombinants that carried a *lucGR* gene coding for a Jamaican click beetle luciferase, which were named MIM945 and A33-945, respectively. The results obtained clearly showed that the strain carrying the prophage had a significantly higher adhesion phenotype, compared to the A33 phage-cured strain (Table 2), thus highlighting that in *S. thermophilus*, likewise in *L. lactis*, the cell wall integrity is correlated with biofilm formation. More recently, *S. thermophilus* biofilm formation was correlated to the presence of the cell-wall protease PrtS which was demonstrated to trigger an aggregative phenotype (Bassi et al., 2017), but this cannot be the case of strain DSM20617^T^ because it is protease-negative (Arioli et al., 2007). In addition, to investigate the role of phage lysin in the *S. thermophilus* chain length, we evaluated the differential sedimentation rates of bacteria according to their chain-forming ability and/or cell size in semi-liquid medium (i.e., liquid M17 – lactose medium containing 0.15% wt/vol agarose). As previously reported in *L. lactis* (Mercier et al., 2002), long chains of *S. thermophilus* stay on the upper part of the semi-liquid culture, while short chain or single cells sediment faster. The comparison between the wild-type and the A33 phage-cured strain (Supplementary Figure S7) revealed that the phage-cured one was characterized by a lower sedimentation capacity in semi-liquid medium (14 ± 2 mm and 24 ± 2 mm for the cured strain and the wild-type, respectively), thus suggesting that its population was primarily represented by cells that were organized into longer chains. Moreover, the wild-type showed faster sedimentation, thus highlighting the presence of shorter chains or single cells in its population. The phase-contrast microscopic analysis of the two cultures confirmed the differences in chain lengths between the two strains (Supplementary Figure S7).

**Table 2:** Adhesion to solid surface of *S. thermophilus* wild-type and the phage-cured derivative A33.

Strain	Adhesion	
	(mO.D. _595__nm_)^a^	(rlu/s)^b^
DSM 20617^T^ wild-type	87 ± 6	/
A33-phage cured	52 ± 4	/
MIM945	/	53935 ± 560
A33-945	/	7716 ± 95


*Adhesion was measured according to the cristal violet protocol (^*a*^) in DSM 20617^*T*^ and A33 strains: the higher mO.D. _*595*__nm_ for DSM 20617^*T*^ reflects its ability to adhere to solid surface; and according to the light emission protocol ( ^*b*^) in recombinant strains MIM945 and A33-945 carrying a *lucGR* gene coding for a Jamaican click beatle luciferase. The light intensity is related to the number of cells adhered on the surface.*

### The Lysogenic *S. thermophilus* Showed Higher Heat Resistance When Compared to the Phage-Cured Derivatives

**FIGURE 6:**
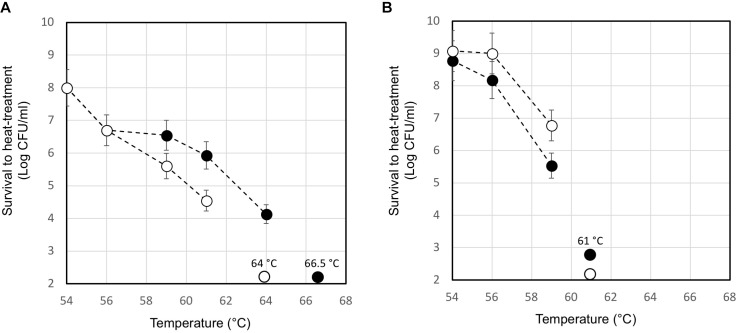
Survival to heat-treatment of cell suspensions of *S. thermophilus* DSM 20617^T^ (black circle) and A33 (white circle) phage-cured derivative. *S. thermophilus* cells have been collected in exponential **(A)** or stationary **(B)** phase of growth, washed and suspended in saline solution at a final concentration of 2 10^9^ event/ml. The data are represented as the average of three independent heat-treatments. The error bars represent the standard deviation. The temperature which affected the survival of each tested strains is indicated.

The moderate but significantly higher level of the *ltaS* transcript measured in the lysogenic strain during exponential growth phase compared to that of A33 phage-cured derivative, prompted us to investigate their heat resistance. Indeed, the properties of teichoic acids in terms of their abilities to bind magnesium ions were demonstrated to be essential for the survival of *S. aureus* under high temperature conditions (Hoover and Gray, 1977). For this purpose, the maximum growth temperature of the lysogenic strain and the phage-cured derivative, was measured. In M17 (2% wt/v lactose) all the strains showed a maximum growth temperature of 45.5°C (no growth was detected at 47.0°C). However, 10 min of heat-treatment in saline solution revealed interesting differences between the wild-type compared to the phage-cured derivative (Figure 6). The heat-treatment resistance assay was performed using cell harvested during the early exponential or stationary phase of growth. When cells were tested in exponential phase, the lysogenic strain showed higher survival to the heat-treatment. Indeed, for DSM 20617^T^ we measured a survival of 1.4 10^4^ CFU/ml after 10 min of exposition to 64°C; no survivals were detected for the phage-cured strains after the same exposition. Indeed, A33 cells were more sensitive to the heat exposition, and they were able to survive only after 10 min of heat treatment at 61°C, displaying survival values of 3.5 10^4^ CFU/ml (Figure 6A). However, cells of lysogenic and phage-cured derivative harvested in the stationary phase of growth appeared more sensitive (Figure 6B). The lower survival to the heat-treatment of autolytic cells collected in stationary phase of growth could be related to the higher cell wall fragility of lysogenic cells (Figure 5). These results were in agreement with the supposed role for lipoteichoic acids in conferring heat-resistance, and with the higher level of *ltaS* gene transcription observed in the exponential growing cells of lysogenic strain compared to the A33 phage-cured strains.

## Conclusion

The study of prophage-dependent adhesion and biofilm formation in *S. thermophilus* could bring new perspectives to the evolutionary role of temperate bacteriophages. Biofilm formation is a mechanism that represents a competitive ecological advantage for microbial strains (Oliveira et al., 2015), more recently the biofilm phenotype of *S. thermophilus* was reported to be a commensal trait that has been lost during the genetic domestication of the species, consistent with its adaptation to the milk environment (Couvigny et al., 2015). In this context, the association between Φ20617 and its host could have been a winning strategy to increase the environmental fitness of this strain. Moreover, we observed a significant decrease in heat resistance in the phage-cured derivative strains. These data could be linked to the observed differences in *ltaS* expression. Despite the fact that the role of lipoteichoic is still not clear, these acids have been implicated in the control of autolysin activity (Höltje and Tomasz, 1975), the provision of a phosphate reserve (Grant, 1979), and cation assimilation (Hoover and Gray, 1977), which is directly linked to thermal resistance (Hoover and Gray, 1977; Fitzgerald and Foster, 2000). When a cell membrane is damaged during sub-lethal heat treatment, large quantities of intracellular and cell wall-bound magnesium will “buffer” the cell against the initial severe effects of heating (Hoover and Gray, 1977). We therefore hypothesize that higher levels of lipoteichoic acids in the wild-type increased the heat resistance due to the prophage 20617 transcriptional modulation. In this context, it is worth of mentioning that strain DSM 2617^T^ has been isolated from pasteurized milk (DSMZ catalogs^4^). The isolation of two phage-cured strain A33 displaying (i) low transcription level of *ltaS* gene and (ii) decrease of heat-resistance, confirmed that phage integration between *eno* and *ltaS* genes should be responsible of the heat-resistance phenotype of the autolytic strain DSM 20617^T^. Therefore, further analysis will be necessary (i) to better understand the role of lipoteichoic acid in heat resistance phenotype of *S. thermophilus*, (ii) to fully address the role of the prophage integration/excision and bacterial autolysin control activity in the autolytic phenotype of *S. thermophilus* DSM 20617^T^. In conclusion, all of the data presented here converge to support new prophage-induced phenotypes in a lysogenic *S. thermophilus*, highlighting the multifactorial constrains that drive a stable association between the prophage and its bacterial host.

## Author Contributions

SA designed, carried out and supervised all the experiments. GE analyzed the prophage genome. GDS, EN, and SC carried out flow cytometry, transcription and thermal sensitivity experiments. AS and MF participated in the writing of the manuscript. DM and SA conceived the project and wrote the manuscript. All authors read and approved the final manuscript.

## Conflict of Interest Statement

The authors declare that the research was conducted in the absence of any commercial or financial relationships that could be construed as a potential conflict of interest.

## Footnotes

1 http://www.ncbi.nlm.nih.gov/gorf/gorf.html

2 http://www.ebi.ac.uk

3 http://web.expasy.org/protparam

4 https://www.dsmz.de/catalogs/catalog-microorganisms.html
