# Supplementary material for: Role of Temperate Bacteriophage ϕ20617 on Streptococcus thermophilus DSM 20617T Autolysis and Biology
Source: Front Microbiol. 2018 Nov 9;9:2719. doi: 10.3389/fmicb.2018.02719 (PMC6237837; doi:10.3389/fmicb.2018.02719)
Supplement: Supplementary file 1 [file Data_Sheet_1.docx]

Manuscript

**Role of temperate bacteriophage *ɸ*20617 on *Streptococcus thermophilus* DSM 20617^T^ autolysis and biology**

Stefania Arioli^1^, Giovanni Eraclio^1,2^, Giulia Della Scala^1,2^, Eros Neri^1,2^, Stefano Colombo^1^, Andrea Scaloni^3^, Maria Grazia Fortina^1^, Diego Mora^1^

*^1^Department of Food Environmental Sciences and Nutrition (DeFENS), University of Milan, Italy;* *^2^Sacco System Srl, Cadorago, Italy; ^3^Proteomics and Mass Spectrometry Laboratory, ISPAAM, National Research Council, 80147 Naples, Italy*

**SUPPLEMENTAL MATERIAL**

**Table S1** Cell lysis of *S. thermophilus* cultures grown in M17 with different concentrations of sucrose, in the presence or absence of the glycolytic inhibitor sodium oxamate.

*Page 3*

**Table S2** Features of the ORFs of *S*. *thermophilus* temperate phage 20617 *Page 4*

**Table S3** RT-qPCR and other PCR primers sequences. *Page 7*

**Figure S1** Growth of S. thermophilus strains in 0.5% wt/vol lactose *Page 8*

**Figure S2** Colonies of *S. thermophilus* wild-type and its derivative mutant A33, cured of *Φ*20617.

*Page 8*

**Figure S3** Growth of S. thermophilus DSM 20617^T^ in microtiter plates using 16 different inoculum levels (from 5 to 2 x 10^6^ events/well), in the presence of 12 increasing concentrations of sucrose (0.05-3%), and in the absence or presence of sodium oxamate *Page 9*

**Figure S4** *S. thermophilus* DSM 20617^T^ genome. *eno*, enolase coding gene, *ltaS*, lipoteichoic acid synthase coding gene, *int*, phage integrase coding genes. Location of PCR primers, and the expected dimension of the relative amplicons, designed for the specific amplification and identification of integration and excision structure are indicated. B) PCR amplification of prophage ORF 48 coding for putative phage tail fiber. Line 1, PCR product obtained using as template DNA from *S. thermophilus* DSM 20617^T^. Line 2, PCR product obtained using as template DNA from A33 phage-cured strain. C) PCR amplification for the identification of integration and excision structures on genomic DNA extracted from *S. thermophilus* DSM 20617^T^ and A33 phage-cured strains. Line 1, 3, 4 and 5 PCR product obtained using as template DNA from *S. thermophilus* DSM 20617^T^ grown in M17 lactose 1%, 0.5%, sucrose 1%, sucrose 0.2% respectively. Line 2, PCR product using as template DNA from *S. thermophilus* A33 phage-cured. M, molecular weight marker. cn, PCR negative control. Expected dimension of PCR fragment are reported. *Page 10*

**Figure S5** qPCR calibration curve obtained using total DNA extracted from *S. thermophilus* A33 and the primer set IntR1-ExcR. *S. thermophilus* cells were quantified by flow cytometry as described in Materials and Methods section. FU, fluorescence units. *Page 11*

**Figure S6** Dot-plots of *S. thermophilus* DSM 20617^T^ and the derivative A33 phage-cured stained with SYBR green I and PI. Live cells were identified in the green gate; cells with membrane damaged were identified in the red gate; dead cells were identified in the blue gate. *S. thermophilus* cells were collected at exponential (O.D._600 nm_ 0.5) and stationary (O.D._600 nm_ 1.5) phase of growth in M17 supplemented with 1% (wt/vol) lactose. The relative abundance (%) of live, damaged and dead population are indicated in each plot.

*Page 12*

**Figure S7** Sedimentation assay for *S. thermophilus* DSM 20617^T^ and the derivative A33 phage-cured *Page 13*

Table S1 Cell lysis of *S. thermophilus* cultures grown in M17 with different concentrations of sucrose, in the presence or absence of the glycolytic inhibitor sodium oxamate.

| Sucrose (%) | Cell lysis (%) | |
| --- | --- | --- |
|  | sodium oxamate (10 mM) | without sodium oxamate |
| 0.2 | 78 | 70 |
| 0.4 | 84 | 44 |
| 0.5 | 64 | 28 |
| 0.6 | 16 | 40 |
| 0.7 | 5 | 12 |

Cells were inoculated at 2 x 10^6^ events/well

Table S2. Features of the ORFs of *S*. *thermophilus* temperate phage 20617^T^.

| **ORF** | **Strand^a^** | | | **Positions**  **Start Stop** | | **Size aa** | **MM^b^ kDa** | | | **pI** | | **Putative RBS and start codon^c^** | **Predicted function^d^** | **Best-match BLASTp result^e^** | **# aa shared with best match / total # aa in best match (% ID)** | **E value** | **Size^f^ (aa)** | **Accession**  **numbers** |
| --- | --- | --- | --- | --- | --- | --- | --- | --- | --- | --- | --- | --- | --- | --- | --- | --- | --- | --- |
| **1** | - | | | 1272 | 103 | 389 | 45 | | | 9.08 | | tAAGGAGat**ATG** | Integrase | H.p., *S. thermophilus* M17PTZA496 | 380/387 (98%) | 0.0 | 387 | [ETW90608.1](http://www.ncbi.nlm.nih.gov/protein/575080352?report=genbank&log$=prottop&blast_rank=2&RID=WGZPEYNA01R) |
| **2** | - | | | 1842 | 1414 | 142 | 15.6 | | | 7.72 | | ggAGGAaatttt**ATG** | Host cell surface-exposed lipoprotein | ORF2, superinfection exclusion lipoprotein, *Streptococcus* phage TP-778L | 141/142 (99%) | 1e-94 | 142 | [YP_008772061.1](http://www.ncbi.nlm.nih.gov/protein/557428125?report=genbank&log$=prottop&blast_rank=2&RID=WH0FG36G015) |
| **3** | - | | | 2290 | 1922 | 122 | 14.7 | | | 5.67 | | gagtagcAAGGAaaagtgaggcttt**ATG** | Helix-turn-helix protein | Hypothetical cI-like repressor, *S*. phage Sfi21 | 117/122 (96%) | 1e-80 | 122 | [NP_049992.1](http://www.ncbi.nlm.nih.gov/protein/9632964?report=genbank&log$=prottop&blast_rank=2&RID=WH6EB9WS015) |
| **4** | - | | | 2647 | 2297 | 116 | 12.7 | | | 6.24 | | AAAGGAaaaaatgc**ATG** | Helix-turn-helix family proteins | cI-like repressor *Streptococcus* phage Sfi21 | 109/116 (94%) | 7e-71 | 127 | [NP_049993.1](http://www.ncbi.nlm.nih.gov/protein/9632965?report=genbank&log$=prottop&blast_rank=2&RID=WHPANMSH01R) |
| **5** | + | | | 2833 | 3060 | 75 | 8.6 | | | 9.10 | | AAAGGAGaTaAcct**ATG** | Helix-turn-helix family proteins | cro repressor, *Streptococcus* phage 5093 | 69/75 (92%) | 9e-43 | 75 | [YP_002925127.1](http://www.ncbi.nlm.nih.gov/protein/238801924?report=genbank&log$=prottop&blast_rank=2&RID=WHUA39B7015) |
| **6** | + | | | 3078 | 3980 | 300 | 34.6 | | | 5.37 | | AAAGGAacTag**ATG** | - | H.p., *Streptococcus* phage 5093 | 262/300 (87%) | 3e-174 | 304 | [YP_002925128.1](http://www.ncbi.nlm.nih.gov/protein/238801925?report=genbank&log$=prottop&blast_rank=2&RID=WKABP6UE01R) |
| **7** | + | | | 3973 | 4521 | 182 | 21.2 | | | 9.24 | | gAAGGAGaaatcataa**ATG** | - | H.p., *S. thermophilus* M17PTZA496 | 158/182 (87%) | 3e-107 | 181 | [ETW90614.1](http://www.ncbi.nlm.nih.gov/protein/575080358?report=genbank&log$=prottop&blast_rank=2&RID=WKBVC5DC014) |
| **8** | + | | | 4527 | 4796 | 89 | 10.7 | | | 9.49 | | AAAGaAatcGggtaacgttt**ATG** | - | Excisionase, *S. thermophilus* M17PTZA496 | 78/79 (99%) | 1e-50 | 79 | [ETW90615.1](http://www.ncbi.nlm.nih.gov/protein/575080359?report=genbank&log$=prottop&blast_rank=2&RID=WKDJ0JJJ014) |
| **9** | + | | | 4774 | 5082 | 102 | 11.7 | | | 7.72 | | cAAGGAGaaaAaac**ATG** | - | H.p., *Streptococcus salivarius* | 79/102 (77%) | 5e-49 | 102 | [WP_014634351.1](http://www.ncbi.nlm.nih.gov/protein/504447249?report=genbank&log$=prottop&blast_rank=2&RID=WKG0CTYJ015) |
| **10** | + | | | 5220 | 5483 | 87 | 10.3 | | | 5.06 | | AAAGGAatTtAa**ATG** | - | ORF p33, *Streptococcus* phage Sfi21 | 68/87 (78%) | 4e-43 | 87 | [NP_597801.1](http://www.ncbi.nlm.nih.gov/protein/19263417?report=genbank&log$=prottop&blast_rank=2&RID=WKGFEF9A01R) |
| **11** | + | | | 5502 | 5660 | 52 | 9.40 | | | 6.56 | | AAAaGAGGaGAaacaaa**ATG** | - | H.p., *S. thermophilus* M17PTZA496 | 47/52 (90%) | 7e-23 | 52 | [ETW90617.1](http://www.ncbi.nlm.nih.gov/protein/575080361?report=genbank&log$=prottop&blast_rank=2&RID=WKH8F4EB014) |
| **12** | + | | | 5877 | 6323 | 148 | 17.6 | | | 8.90 | | tAtGGAGaTtAtaacataa**ATG** | Phage replisome | Replisome, *S.salivarius* | 114/148 (77%) | 5e-75 | 273 | [WP_037597577.1](http://www.ncbi.nlm.nih.gov/protein/739744631?report=genbank&log$=prottop&blast_rank=2&RID=WKHZ51K401R) |
| **13** | + | | | 6296 | 6724 | 142 | 16.3 | | | 4.78 | | AAAttgGacaAtgtccagccg**ATG** | DNA replication p. | Replisome organizer, *S. equi* | 90/135 (67%) | 4e-55 | 269 | [WP_050316415.1](http://www.ncbi.nlm.nih.gov/protein/912942714?report=genbank&log$=prottop&blast_rank=2&RID=WKJCJXMT01R) |
| **14** | + | | | 6737 | 7519 | 260 | 30.3 | | | 8.54 | | tAAGGAGtcacttct**ATG** | DNA replication | DnaC-like protein, *S*. phage Abc2 | 239/260 (92%) | 2e-170 | 260 | YP_003347440.1 |
| **15** | | + | 7516 | | 7698 | 60 | | 7.21 | 5.57 | | AgAGGAtGatgct**ATG** | | - | ORF6a *Streptococcus* phage 7201 | 59/60 (98%) | 8e-34 | 60 | NP_038306.1 |
| **16** | | + | 7824 | | 8480 | 218 | | 25 | 6.23 | | AAAGGgaGacAac**ATG** | | DNA single strand p. | ORF7, *Streptococcus* phage 7201 | 216/218 (99%) | 4e-157 | 218 | [NP_038308.1](http://www.ncbi.nlm.nih.gov/protein/9634634?report=genbank&log$=prottop&blast_rank=2&RID=WKNE0JNC014) |
| **17** | | + | 8483 | | 9442 | 319 | | 37.5 | 5.41 | | AAcGGAaGgGttaaat**ATG** | | - | ORF34, *Streptococcus* phage Abc2 | 291/318(92%) | 0.0 | 323 | [YP_003347443.1](http://www.ncbi.nlm.nih.gov/protein/281416419?report=genbank&log$=prottop&blast_rank=2&RID=WKTHFND3014) |
| **18** | | + | 9463 | | 9927 | 154 | | 17 | 6.31 | | AgAGGAGaaaAaac**ATG** | | DNA single strand p. | Ss DNA binding p., *Streptococcus* phage 5093 | 130/154 (84%) | 2e-86 | 150 | [YP_002925089.1](http://www.ncbi.nlm.nih.gov/protein/238801886?report=genbank&log$=prottop&blast_rank=2&RID=WKUP8JJ6015) |
| **19** | | + | 9937 | | 10398 | 153 | | 18 | 9.33 | | ccattctaatatactgct**ATG** | | Resolvase | Endodeoxyribonuclease, *Streptococcus* phage 5093 | 143/153 (93%) | 1e-100 | 153 | [YP_002925090.1](http://www.ncbi.nlm.nih.gov/protein/238801887?report=genbank&log$=prottop&blast_rank=2&RID=WKVFJU6P015) |
| **20** | | + | 10395 | | 10634 | 79 | | 9.24 | 5.27 | | cAAGGAGcTGga**ATG** | | - | ORF38, h.p., *Streptococcus* phage Sfi19 | 65/79 (82%) | 3e-40 | 89 | [NP_049958.1](http://www.ncbi.nlm.nih.gov/protein/9632929?report=genbank&log$=prottop&blast_rank=2&RID=WKWE7ADT014) |
| **21** | | + | 10622 | | 10795 | 57 | | 6.42 | 9.61 | | AAAGGAGaTGggagaatt**ATG** | | - | ORF17, h.p., *Streptococcus* phage TP-J34 | 46/55 (84%) | 4e-26 | 56 | [YP_007392265.1](http://www.ncbi.nlm.nih.gov/protein/444475892?report=genbank&log$=prottop&blast_rank=2&RID=WKWW2TB001R) |
| **22** | | + | 10792 | | 10953 | 53 | | 6.58 | 5.84 | | AAAGaGatTagtagagtt**ATG** | | - | H.p., *Streptococcus thermophilus* M17PTZA496 | 42/49 (86%) | 9e-21 | 52 | ETW90629.1 |
| **23** | | + | 11102 | | 11184 | 60 | | 7.54 | 6.27 | | AAAcGAaGaGgtagaaaa**ATG** | | - | H.p., *Streptococcus macedonicus* | 58/60 (97%) | 3e-30 | 60 | WP_039670732.1 |
| **24** | | + | 11312 | | 11926 | 204 | | 24 | 5.27 | | AAAGcAccTtAtaaaa**ATG** | | - | H.p., *Streptococcus thermophilus* M17PTZA496 | 96/202(48%) | 8e-44 | 193 | ETW90635.1 |
| **25** | | + | 11910 | | 12026 | 38 | | 4.61 | 4.29 | | AAAGagGtgGAaa**ATG** | | - | H.p., *Streptococcus agalactiae* | 25/30 (83%) | 2e-09 | 115 | [WP_017770149.1](http://www.ncbi.nlm.nih.gov/protein/516380116?report=genbank&log$=prottop&blast_rank=2&RID=WM4HFKY2015) |
| **26** | | + | 12047 | | 12550 | 167 | | 19.6 | 5.17 | | AAAGtGAGGaatat**ATG** | | - | H.p., *Streptococcus mitis* | 80/175 (46%) | 2e-35 | 168 | [WP_001025454.1](http://www.ncbi.nlm.nih.gov/protein/446948198?report=genbank&log$=prottop&blast_rank=2&RID=WM4YKKDV01R) |
| **27** | | + | 12551 | | 12973 | 140 | | 15.8 | 5.73 | | AgAaGAGGTGAagtaa**GTG** | | Transcriptional regulator | DNA binding protein, *Streptococcus* phage 2972 | 81/173 (47%) | 5e-38 | 170 | [YP_238525.1](http://www.ncbi.nlm.nih.gov/protein/66391801?report=genbank&log$=prottop&blast_rank=2&RID=WM5FC60B015) |
| **28** | | + | 12942 | | 13250 | 102 | | 11.3 | 9.79 | | AAAGGgaagatgataa**ATG** | | - | H.p., *Streptococcus salivarius* | 77/102 (75%) | 3e-50 | 105 | WP_038675626.1 |
| **29** | | + | 13247 | | 13681 | 144 | | 16.9 | 4.41 | | AAAGGAaGaGgaca**ATG** | | YopX protein | H.p., Streptococcus phage YMC-2011 | 85/141 (60%) | 1e-51 | 129 | WP_045002244.1 |
| **30** | | + | 13678 | | 13926 | 82 | | 9.73 | 9.48 | | agaattAttaGAGGaactgaa**ATG** | | - | H.p., *Streptococcus pneumoniae* | 35/71 (49%) | 3e-13 | 82 | [CIS16940.1](http://www.ncbi.nlm.nih.gov/protein/810707897?report=genbank&log$=prottop&blast_rank=2&RID=WM78VWUF014) |
| **31** | | + | 13996 | | 14703 | 235 | | 27.7 | 9.37 | | tgAGGAGtTGttaagcc**ATG** | | - | ORF45, h.p., *Streptococcus* phage Sfi19 | 205/235 (87%) | 5e-146 | 235 | NP_049965.1 |
| **32** | | + | 14916 | | 15332 | 138 | | 16.1 | 7.84 | | aaAAAGGgGtcGAata**ATG** | | Transcriptional regulator | ORF1, h.p., *Streptococcus* phage 2972 | 131/138 (95%) | 4e-89 | 137 | [YP_238484.1](http://www.ncbi.nlm.nih.gov/protein/66391760?report=genbank&log$=prottop&blast_rank=2&RID=WRAWCHHB014) |
| **33** | | + | 15514 | | 15966 | 150 | | 16.8 | 6.01 | | aaatatttGcgGaTGAatac**TTG** | | Terminase small subunit | ORF1, *Streptococcus* phage Alq132 | 149/150 (99%) | 2e-100 | 150 | [YP_003344847.1](http://www.ncbi.nlm.nih.gov/protein/273809772?report=genbank&log$=prottop&blast_rank=2&RID=WRBFS9N3014) |
| **34** | | + | 15953 | | 17188 | 411 | | 47 | 5.77 | | AAAGGAGcTGtaagcg**ATG** | | Terminase large subunit | ORF29, *Streptococcus* phage TP-J34 | 400/411 (97%) | 0.0 | 411 | [YP_007392276.1](http://www.ncbi.nlm.nih.gov/protein/444475903?report=genbank&log$=prottop&blast_rank=2&RID=WRBUWZVX014) |
| **35** | | + | 17197 | | 18696 | 499 | | 56.6 | 4.80 | | agttAGGAGaAGtg**ATG** | | Portal protein | PP, *S. thermophilus* M17PTZA496 | 451/486 (93%) | 0.0 | 501 | [ETW90644.1](http://www.ncbi.nlm.nih.gov/protein/575080388?report=genbank&log$=prottop&blast_rank=2&RID=WRCB9BZ2015) |
| **36** | | + | 18700 | | 19593 | 297 | | 34.4 | 9.45 | | aatgccgcGGAGGaGtaacgt**ATG** | | Head morphogenesis p. | ORF4, capsid, *Streptococcus* phage Alq132 | 206/297 (69%) | 1e-140 | 297 | YP_003344850.1 |
| **37** | | + | 19775 | | 20368 | 197 | | 21.6 | 4.69 | | tagAtAGGAGaacAaa**ATG** | | - | ORF5, scaffold protein, *Streptococcus* phage Alq132 | 181/197 (92%) | 8e-123 | 193 | [YP_003344851.1](http://www.ncbi.nlm.nih.gov/protein/273809732?report=genbank&log$=prottop&blast_rank=2&RID=WSKB9EK8015) |
| **38** | | + | 20385 | | 20744 | 119 | | 12.8 | 7.82 | | AAAGGAaagGgaaat**ATG** | | - | ORF6, capsid, *Streptococcus* phage Alq132 | 100/119 (84%) | 1e-62 | 119 | YP_003344852.1 |
| **39** | | + | 20767 | | 21813 | 348 | | 37.4 | 4.98 | | AAAGGgGGactattaaaac**ATG** | | Major capsid protein | ORF10, *Streptococcus* phage 858 | 323/348 (93%) | 0.0 | 348 | YP_001686804.1 |
| **40** | | + | 21829 | | 21984 | 51 | | 5.91 | 9.44 | | AgAGGAtaTcgat**ATG** | | - | H.p., *Streptococcus thermophilus* M17PTZA496 | 26/38 (68%) | 1e-08 | 53 | [ETW90649.1](http://www.ncbi.nlm.nih.gov/protein/575080393?report=genbank&log$=prottop&blast_rank=2&RID=WSP6WU0M015) |
| **41** | | + | 22002 | | 22346 | 114 | | 13.4 | 4.51 | | aatagcAaGAaaTGAggcggat**ATG** | | Head-tail connector p. | ORF113, *Streptococcus* phage Sfi11 | 99/110 (90%) | 2e-64 | 113 | [NP_056682.1](http://www.ncbi.nlm.nih.gov/protein/9635017?report=genbank&log$=prottop&blast_rank=2&RID=WSR7BUBH015) |
| **42** | | + | 22343 | | 22657 | 104 | | 11.7 | 9.79 | | AAAGagGGaGAggtgctatttct**ATG** | | - | ORF104, *Streptococcus* phage Sfi11 | 75/103 (73%) | 1e-46 | 104 | [NP_056683.1](http://www.ncbi.nlm.nih.gov/protein/9635018?report=genbank&log$=prottop&blast_rank=2&RID=WSSK1XE2015) |
| **43** | | + | 22657 | | 22995 | 112 | | 12.4 | 9.19 | | AAAcagGGTGgttggtta**ATG** | | Putative tail component | ORF13, h.p., *Streptococcus* phage 2972 | 107/112 (96%) | 1e-70 | 112 | YP_238496.1 |
| **44** | | + | 22997 | | 23383 | 128 | | 14.5 | 4.84 | | gAAAtGgctaagtgGGAataag**ATG** | | - | ORF14, h.p., *Streptococcus* phage 2972 | 115/128 (90%) | 2e-80 | 128 | YP_238497.1 |
| **45** | | + | 23395 | | 23901 | 168 | | 18.5 | 4.80 | | AAAGGAGGaaAac**ATG** | | Major tail protein | ORF37, h.p., *Streptococcus* phage TP-778L | 144/161 (89%) | 7e-99 | 169 | YP_008772096.1 |
| **46** | | + | 23979 | | 24332 | 117 | | 13.1 | 4.66 | | AtAGGAGtTaAaaaca**ATG** | | Tail assembly chaperone protein | ORF14, h.p., *Streptococcu*s phage Alq132 | 116/117 (99%) | 1e-77 | 117 | YP_003344860.1 |
| **47** | | + | 24383 | | 24700 | 105 | | 12.7 | 10.12 | | acgAGGAatTaATcactaatgct**ATG** | | - | ORF39, h.p., *Streptococcus* phage O1205 | 98/105 (93%) | 6e-62 | 105 | NP_695117 |
| **48** | | + | 24690 | | 29306 | 1538 | | 155.4 | 9.56 | | AAAGagGGTtgctag**ATG** | | Tape protein | Tail protein, *Streptococcus thermophilus* M17PTZA496 | 1353/1545 (88%) | 0.0 | 1545 | ETW90657 |
| **49** | | + | 29306 | | 30841 | 511 | | 57.7 | 5.39 | | aaacgtAtgaGAGGTattaaata**ATG** | | Tail protein | ORF20, *Streptococcus* phage 858 | 489/511 (96%) | 0.0 | 511 | YP_001686814 |
| **50** | | + | 30841 | | 34275 | 1144 | | 126.6 | 5.33 | | AAgaaAGGTatttgtA**ATG** | | Structural protein | ORF18, receptor-binding protein, *Streptococcus* phage Alq132 | 862/1191 (72%) | 0.0 | 1168 | YP_003344864 |
| **51** | | + | 34278 | | 36287 | 669 | | 74.2 | 6.23 | | gtgGGAGGTatttaata**ATG** | | Structural protein | ORF39, h.p., *Streptococcus* phage 7201 | 510/669 (76%) | 0.0 | 669 | NP_038340 |
| **52** | | + | 36303 | | 36725 | 140 | | 15.9 | 4.41 | | agaGAAGGaaAattc**ATG** | | - | ORF20, h.p., *Streptococcus* phage Alq132 | 110/134 (82%) | 2e-71 | 136 | YP_003344866.1 |
| **53** | | + | 36749 | | 36895 | 48 | | 5.42 | 9.70 | | aaagAAGGAaaaaAat**ATG** | | - | ORF51, h.p., Streptococcus phage TP-J34 | 41/48 (85%) | 8e-20 | 48 | YP_007392298 |
| **54** | | + | 36909 | | 37232 | 107 | | 12.5 | 6.42 | | caAgAatAGtTAGgagtgcggt**ATG** | | - | H.p., *Streptococcus thermophilus* M17PTZA496 | 100/107 (93%) | 5e-66 | 107 | ETW90663 |
| **55** | | + | 37241 | | 37483 | 80 | | 8.80 | 8.09 | | AgAGGAtGactaataaa**ATG** | | Holin | ORF24, holin, *Streptococcus* phage DT1 | 71/80 (89%) | 9e-43 | 80 | NP_049412 |
| **56** | | + | 37485 | | 38330 | 281 | | 31 | 4.25 | | tAAGGAaGaaAaataat**ATG** | | Lysin | ORF44, *Streptococcus* phage 7201 | 255/281(91%) | 7e-180 | 281 | NP_038345.1 |
| **57** | | + | 38815 | | 39159 | 114 | | 13.2 | 9.87 | | AAAGagaGgGAataaaagat**ATG** | | - | H.p., *Streptococcus thermophilus* M17PTZA496 | 111/114 (97%) | 3e-74 | 114 | ETW90666 |
| **58** | | + | 39181 | | 39732 | 183 | | 21.3 | 6.61 | | aaagAAGGAGaaataaaa**ATG** | | - | ORF183, h.p., *Streptococcus* phage Sfi11 | 163/183 (89%) | 5e-117 | 183 | NP_056701 |
| **59** | | + | 39758 | | 40009 | 83 | | 9.80 | 4.78 | | AAAcGAGGTaAaaaca**ATG** | | - | H.p., *Streptococcus* phage 5093 | 81/83 (98%) | 3e-50 | 86 | YP_002925121 |
| **60** | | + | 40035 | | 40214 | 59 | | 7.32 | 9.40 | | atagAAAGGtttaacTGAt**ATG** | | - | H.p., *Streptococcus* phage 5093 | 55/59 (93%) | 6e-32 | 59 | YP_002925122 |
| **61** | | + | 40273 | | 40695 | 140 | | 16.3 | 4.36 | | AgAGGAaaTaAatgaa**ATG** | | - | ORF60, h.p., *Streptococcus* phage TP-J34 | 120/140 (86%) | 3e-78 | 140 | YP_007392307 |

H.p., hypothetical protein; DNA replication p., DNA replication protein; PP, *S. thermophilus*, portal protein *S. thermophilus*; head-tail connector p., head tail connector protein. ^a^ Orientation of the gene in the genome. ^b^ MM, molecular mass. ^c^ RBS, ribosomal binding site: uppercase letters represent the hypothetical RBS sequences, bold letters the starting codons. ^d^ – indicates no significant matches. ^e^Second best match.  ^f^ Total size of the aligned proteins.

Table S3. RT-qPCR and other PCR primers sequences

| Gene target or chromosomal locus | Primers set |
| --- | --- |
| *pgk* | pgkF (5’-gttaccgatttcgataccttgcgc-3’)  pgkR (5’-tccagaacgtccattcgtggctat-3’) |
| *eno* | enoF (5’-caaggtattgaccgcgca-3’)  enoR (5’-tggaaagcgattggagcg-3’) |
| *ltaS* | ltaSF (5’-ggaggtcttgtcccatctgg-3’)  ltaSR (5’-ggtttcccactcgctgacac-3’) |
| *ureC* | ureCF (5’-gctgacggtagtaatgcaacaa-3’)  ureCR (5’-gtgtgtcttaattcctgctgca-3’) |
| ORF 48 (phage tale fiber) | OTY1 (5’-agggtgttctcggtgcta-3’)  OTY2 (5’-ccgcaaagtcctctgtct-3’) |
| ORF 55 (holin) | holF (5’-AGTAGCTCTTGTCTCGGC-3’)  holR (5’-caatgctcgtccactgtc-3’) |
| ORF 56 (lysin) | lysF (5’-tgtcacaatctggcggag-3’)  lyrR (5’-gttgttggtttgcggtgc-3’) |
| Prophage-host integration structure | IntR1 (5’-CGAAAACGACTGGGACGG-3’)  IntR2 (5’-CATGAAAGACCAAGCCGTC-3’) |
| host excision structure | IntR1 (5’-CGAAAACGACTGGGACGG-3’)  ExcR (5’-CTAAGCCTAAATTCCCCC-3’) |

All RT-qPCR primers were designed to work at an annealing temperature of 61 °C. The optimal annealing temperature was tested for all the primer sets using a temperature gradient PCR protocol ranging from 57 to 72 °C. The amplification efficiency (%), tested using serial dilution of quantified DNA extracted from *S. thermophilus* DSMZ 20617^T^, was between 95 and 100% for all the primer sets.
